# Liquid Biopsy Biomarkers in Bladder Cancer: A Current Need for Patient Diagnosis and Monitoring

**DOI:** 10.3390/ijms19092514

**Published:** 2018-08-24

**Authors:** Iris Lodewijk, Marta Dueñas, Carolina Rubio, Ester Munera-Maravilla, Cristina Segovia, Alejandra Bernardini, Alicia Teijeira, Jesús M. Paramio, Cristian Suárez-Cabrera

**Affiliations:** 1 Molecular Oncology Unit, CIEMAT (Centro de Investigaciones Energéticas, Medioambientales y Tecnológicas), Avenida Complutense nº 40, 28040 Madrid, Spain; IrisAdriana.Lodewijk@externos.ciemat.es (I.L.); marta.duenas@ciemat.es (M.D.); carolina.rubio@externos.ciemat.es (C.R.); ester.munera@ciemat.es (E.M.-M.); cristina.segovia@ciemat.es (C.S.); Alejandra.bernardini@externos.ciemat.es (A.B.); aliciateijeira.merced@gmail.com (A.T.); jesusm.paramio@ciemat.es (J.M.P.); 2 Biomedical Research Institute I+12, University Hospital “12 de Octubre”, Av Córdoba s/n, 28041 Madrid, Spain; 3 Centro de Investigación Biomédica en Red de Cáncer (CIBERONC), 28029 Madrid, Spain

**Keywords:** bladder cancer, liquid biopsy, biomarkers

## Abstract

Bladder Cancer (BC) represents a clinical and social challenge due to its high incidence and recurrence rates, as well as the limited advances in effective disease management. Currently, a combination of cytology and cystoscopy is the routinely used methodology for diagnosis, prognosis and disease surveillance. However, both the poor sensitivity of cytology tests as well as the high invasiveness and big variation in tumour stage and grade interpretation using cystoscopy, emphasizes the urgent need for improvements in BC clinical guidance. Liquid biopsy represents a new non-invasive approach that has been extensively studied over the last decade and holds great promise. Even though its clinical use is still compromised, multiple studies have recently focused on the potential application of biomarkers in liquid biopsies for BC, including circulating tumour cells and DNA, RNAs, proteins and peptides, metabolites and extracellular vesicles. In this review, we summarize the present knowledge on the different types of biomarkers, their potential use in liquid biopsy and clinical applications in BC.

## 1. Introduction: Bladder Cancer Issues and Liquid Biopsy

Bladder cancer (BC) is the most common malignancy of the urinary tract, representing a highly prevalent disease which affects primarily elderly people. For both sexes combined, it is the 9th most common cancer diagnosed worldwide and a significant cause of tumour-related death, with an estimated 165,000 deaths per year [1]. BC represents an important health problem with an age-standardized incidence rate (per 100,000 person-years) of 9 in men versus 2.2 in women and an age-standardized mortality rate (per 100,000 person-years) of 3.2 and 0.9, respectively [2,3,4]. The incidence and mortality rate are stagnant due to the scarcity of newly developed effective treatments and options for prevention [5,6].

BC can be divided in two major classes based on tumour stage, I) non-muscle invasive bladder cancer (NMIBC), which is either confined to the urothelium (carcinoma in situ (CIS)-or stage Ta, 5-year survival rate of 95.4%) or the lamina propia (stage T1, 5-year survival rate of approximately 88%) and II) muscle-invasive bladder cancer (MIBC) (stage T2, T3 and T4, representing 5-year survival rates of 69.4%, 34.9% and 4.8%, respectively) [7,8]. NMIBC represents the most frequent form of BC, presented by approximately 70–80% of patients at diagnosis and is primarily treated by transurethral resection of the bladder tumour (TURBT), which is considered fundamental for the diagnosis and prognosis of the disease [9,10]. Dependent upon certain pathological characteristics (e.g., size and number of implants), TURBT is followed by intravesical instillation with chemotherapeutics, such as mitomycin, or the immunotherapeutic Bacillus Calmette-Guérin (BCG) [11,12]. However, despite TURBT and chemo/immunotherapy as first-line treatment, NMIBC displays a high recurrence incidence (50–70%) with tumour progression towards invasive tumours in at least 10–15% of the cases, due to minimal residual disease (MRD) which remained undetected [9,10]. The extraordinary rates of recurrence and the likelihood to progress require continuous follow-up of NMIBC patients by cystoscopy (every 3–6 months during the next 5 years) and urine cytology, making NMIBC one of the most costly malignancies for the National Health systems of developed countries [11,13]. Accordingly, BC represents the most expensive human cancer from diagnosis to death, with an estimated cost of $187,000 per patient in the United States [14]. In 2010, its total annual cost was estimated at $4 billion, which is expected to rise to approximately $5 billion by 2020 [14,15]. In the European Union, in 2012, the total BC expenditure has been determined at €4.9 billion, with health care accounting for €2.9 billion (59%) [16].

The remaining 20–30% of BC patients presents MIBC at diagnosis. Once tumour progression is observed the prognosis declines [17,18]. Treatment of invasive BC currently consists of radical cystectomy followed by platin-based chemotherapy. Nevertheless, clinical benefit of the addition of neoadjuvant chemotherapy (NAC) (like cisplatin, methotrexate, vinblastine and gemcitabine) has been evaluated by several studies [19,20,21]. NAC is presumed to diminish the burden of micrometastatic disease and can be used to predict chemosensitivity of the tumour [2]. Despite conflicting results shown by multiple randomized phase III trials (due to differences in for example, chemotherapy used, number of cycles and trial design), a significant survival benefit in favour of NAC has been indicated by various meta-analyses [19,20]. Unfortunately, metastatic spreading remains an important problem in a high fraction of the cases (50–70%), resulting in very low survival rates (5-year survival rate of 4.8%) [2,8,22].

Despite multiple trials, no new effective therapeutic options have been developed throughout the last decades [23], with the exception of immunotherapy based on checkpoint inhibitors. Even though these checkpoint inhibitors have shown promising results in patients with advanced or recurrent BC, only 20–35% of the BC patients benefit from this therapy and overall survival is still limited [24,25].

The typical and most important clinical indication for BC is haematuria. Nowadays, a combination of urine cytology and cystoscopy is still the routinely used methodology by excellence for detection, diagnosis and surveillance of this disease. Cytology remains the gold standard for detection of urothelial carcinoma. BC urinary cytology shows a specificity of approximately 98% and a sensitivity of 38% [26] (Table 1). However, the sensitivity of this test significantly increases with malignancy grade, reaching a reasonable sensitivity of >60% for CIS and high-grade lesions [26,27]. In 1997, in order to improve cytology predictive values, Fradet and Lockhard developed an immunofluorescence test (uCyt+) which was based on detection of three BC antigens (M344, LDQ10 and 19A11) in exfoliated cells [28], improving the sensitivity of cytology to approximately 73% but decreasing the specificity to 66% due to the requirement of a large number of exfoliated cells [29] (Table 1). Cystoscopy is currently the gold standard technique in clinical practice for detection and follow-up of BC, achieving a sensitivity of approximately 85–90% and 65–70% to detect exophytic tumours and CIS, respectively [27,30,31,32,33]. Nevertheless, this procedure is highly invasive, showing a big inter-observer and intra-observer variation in the tumour stage and grade interpretation [27,30,31,32,33].

**Figure 1:**
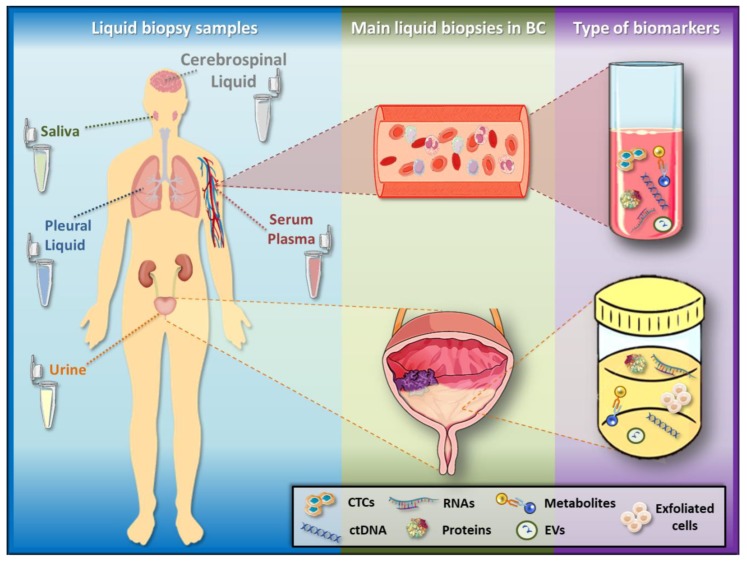
Liquid biopsy samples and biomarkers. Liquid biopsy samples include urine, serum, plasma, saliva, cerebrospinal and pleural fluid, among others. In BC, the liquid biopsies more widely used as detection and surveillance systems are urine (by its intimate contact with the tumour), as well as serum and plasma, which allow the follow-up of advanced disease. These liquid biopsies present several biomarkers, such as circulating tumour cells (CTCs), circulating cell-free tumour DNA (ctDNA), RNAs, proteins, metabolites and extracellular vesicles (EVs). Additionally, exfoliated cells derived from a tumour can be found in urine.

Therefore, it is clear that there is an urgent need for improvements in diagnosis, prognosis and follow-up of BC patients. Over the last decades, tumour biopsies have revealed details with regard to the genetic profile of tumours, allowing the prediction of prognosis, tumour progression as well as therapy response and resistance [34]. Recently, the potential use of liquid biopsy as a new non-invasive way to determine the genomic landscape of cancer patients, screen treatment response, quantify MRD and assess therapy resistance is gaining significant attention [34,35,36,37,38,39,40]. The term “liquid biopsy” means the sampling and analysis of biological fluids, including blood, plasma, urine, pleural liquid, cerebrospinal fluid and saliva (Figure 1) [36,39]. The analysis is based on different cells and molecules which can be obtained from liquid biopsies: circulating tumour cells (CTCs), circulating cell-free tumour DNA (ctDNA), messenger RNAs (mRNAs), micro-RNAs (miRNAs), long non-coding RNAs (lncRNAs), proteins and peptides, metabolites and vesicles (exosomes and endosomes) (Figure 1). Even though the presence of circulating free DNA and RNA in human blood was first demonstrated in 1948 [41], only a few liquid biopsies are currently approved for clinical use. In recent years, cancer research has been mainly focused on the introduction of suitable biomarkers, indicating the presence, recurrence and progression of a disease, as well as the appropriated treatment for a specific type of cancer. Taken together, biomarkers present in liquid biopsies hold great promise, as they are able to record and monitor the disease stage at real time and predict prognosis, recurrence, therapy response and resistance, without invasive intervention. 

Thus, the potential use of liquid biopsy as a new non-invasive approach to improve BC management is far reaching. Even though their extensive applications are only starting to emerge in clinical practice, multiple studies have indicated the potential use of different biomarkers in liquid biopsies for BC. In this review, we provide an overview of the more important studies regarding the different types of biomarkers in liquid biopsy and their clinical applications in BC.

## 2. Liquid Biopsy Biomarkers and Their Clinical Applications

### 2.1. Circulating Tumour Cells (CTCs)

CTCs were first discovered in breast cancer patients in 1869 by Ashworth and colleagues [54]. They are tumour cells of approximately 4 to 50 μm, which are being released from the tumour site into the bloodstream, thereby representing the main mechanism for metastasis [54,55]. CTC detection systems emerged from the need to find new methods to detect early metastatic disease in a less invasive way compared to conventional methods currently available, such as radiological evaluation. In recent years, a wide variety of approaches has been developed for the detection of CTCs, some of which have been implemented in clinical practice. These techniques include immunocytochemistry, reverse-transcriptase polymerase chain reaction, flow cytometry and the CellSearch system, which is the only approach approved by the USA Food and Drug Administration (FDA) [56].

In certain types of solid tumours, such as breast, colorectal cancer and gastric tumours, it has been reported that the presence of CTCs is an indicator of poor prognosis [57,58,59,60]. In BC, the presence of CTCs has also been proposed to be associated with a bad prognosis and the amount of CTCs found in blood has been indicated to correlate with short disease-free survival in metastatic BC [61]. However, the relevance for NMIBC is still controversial.

#### 2.1.1. CTC Detection Methods

**Figure 2:**
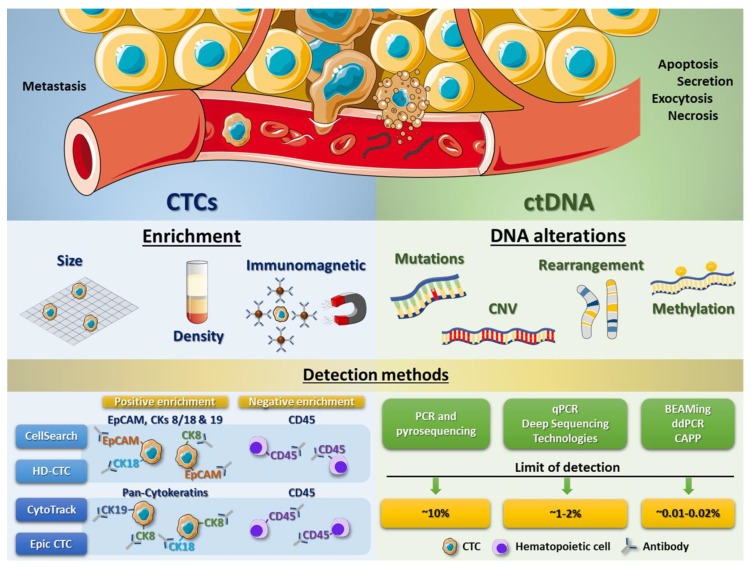
CTC and ctDNA processing methods. Scheme showing some enrichment techniques to isolate CTCs from peripheral blood cells (erythrocytes and leukocytes) and different detection systems based on immunomagnetic assays, using specific antibodies to recognize antigens present in tumour cells (like EpCAM or cytokeratins) as well as to exclude leukocytes (using antibodies against CD45) (left panel). Right panel displays the different DNA alterations (including mutations, copy number variations (CNVs), gene rearrangements or methylation variations) which can be analysed from ctDNA, as well as different detection methods and their correspondent limit of detection.

Since CTCs are very rare and the amount of cells available is around 1 to 10 in 10^6^–10^8^ white blood cells, their detection, enumeration and molecular characterization is a challenge [62]. Accordingly, an efficient and reliable method for both isolation and characterization of these cells is needed [62,63]. Nowadays, different isolation techniques have been developed, all of which have a first enrichment step before the cells can finally be analysed. Enrichment can be carried out by different methods, including techniques based on physical properties such as size (by microfilters that isolate CTCs regarding to their greater size), density or deformability, as well as on biological properties of CTCs (for example, using immunomagnetic assays) (Figure 2) (reviewed in [64]). Immunomagnetic enrichment can be either negative or positive, both of which are available for in vivo assays, enabling a better sample analysis. Whereas negative enrichment does not rely on the biomarker expression of CTCs but on markers of hematopoietic cells (like CD45, a leukocytic antigen) and allows collection of cells in their intact form (depleting most of the leukocytes and erythrocytes), positive enrichment (using specific CTC biomarkers) has its own advantages including a low false-positive CTC detection rate (Figure 2) (reviewed in [64]).

The CellSearch system (Veridex, LLC, Warren, NJ, USA) is one of those technologies developed and, in this case, approved by the FDA for the isolation of CTCs (Table 1). CellSearch CTC Test is based on immunomagnetic enrichment and, initially, permitted enumeration of CTCs of epithelial origin by targeting only EpCAM for capturing CTCs. However, some studies have pointed out the difficulty in obtaining sufficient EpCAM-expressing CTCs from patients with advanced disease to reach statistically significant conclusions from a study or clinical trial [65]. Therefore, the recent versions of this test also select CTCs by other surface proteins, selecting those cells that are CD45−, EpCAM+ and cytokeratin 8/18+ and/or 19+. Though CellSearch is the most frequently used and still the gold standard today, new ways to detect CTCs have come up recently. CytoTrack is a similar method which allows detection and quantification of CTCs using a scanning fluorescence microscope [66]. For this test, a cocktail of a range of cytokeratins (pan-cytokeratin antibody) and CD45 (to deplete blood cells) is used [66]. Given that CytoTrack relies on a cytokeratin signal to detect cells and CellSearch depends on both EpCAM and cytokeratin expression, these two different approaches could give rise to significantly different results with regard to CTC detection. The advantage of Cytotrack is the possibility of staining with different antibodies which allows identification of new CTC biomarkers [67]. For example, HER2, which is considered a breast cancer biomarker, has also been used as target antigen for this technique [68,69]. By performing a comparative analysis between both systems, CellSearch and Cytotrack, Hillig et al. found that the two CTC technologies have similar recovery of cells spiked into blood (69% vs. 71%, *p* = 0.58, respectively) [67]. However, CellSearch shows a lower variability in the analysis [67]. Another promising method is the Epic CTC Platform, whose detection system is based on the use of cytokeratins as CTC biomarkers and CD45 as hematopoietic marker. Even though this approach is similar to CytoTrack, the Epic CTC platform also integrates downstream capabilities for the evaluation of cell morphology characteristics, protein biomarker expression and genomic analyses (Fluorescence In Situ Hybridization (FISH) and Next-Generation Sequencing (NGS)) [70]. In an analytical validation of the Epic CTC Platform capabilities, Werner et al. assayed the performance, including accuracy, linearity, specificity and intra/inter-assay precision, of CTC enumeration in healthy donor blood samples spiked with varying concentrations of cancer cell line controls [71]. They found a high percentage of nucleated cell recovery for all cancer cell concentrations tested and showed excellent assay linearity (*R*^2^ = 0.999). Besides, using a small cohort of metastatic castration-resistant prostate cancer patient samples tested with the Epic CTC Platform, detection of ≥1 traditional CTC/mL in 89% of patient samples was shown, whereas 100% of the cancer patient samples had ≥1 CTC/mL when additionally considering the cytokeratin negative and apoptotic CTC subpopulations, compared to healthy donor samples (in which zero CTCs were enumerated in all 18 samples) (Figure 2) [70].

An improved CellSearch method is HD-CTC (from High Definition CTC; Epic Sciences, Inc., San Diego, CA, USA), which is not only based on EpCAM, cytokeratins and CD45 immunofluorescence staining but also on morphological characterization, size and high throughput counting, allowing the identification of apoptotic cells by DAPI staining and imaging using a high definition scanner (Figure 2). This detection method has been demonstrated to be more sensitive than the original CellSearch [71]. Additional to previously described detection methods, many other approaches have been developed over the last years by multiple commercial laboratories, evidencing the great potential of CTC detection at present and in future clinical procedures (reviewed in [64]).

However, these detection systems usually use small volumes of peripheral blood (<10 mL), showing a yield of 0.1–0.2% with respect to all tumour cells present in whole blood [72]. To overcome this problem in CTC detection and to evaluate large blood volumes, some groups have been exploring the potential of apheresis as CTC isolation method previous to the use of detection systems. In breast and pancreatic cancer patients, apheresis has demonstrated to improve the recovery of CTCs, showing better yield than the CellSearch system [73,74]. Besides, since CTCs probably have representative features of primary tumours, obtaining a sufficient number of CTCs could depict a global view of the tumour alterations and would allow carrying out different genomic analyses in order to define tumour and metastasis features. At present, there is a European Consortium “CTC Therapeutic Apheresis: CTCTrap project” (http://www.utwente.nl/en/tnw/ctctrap/) focused on improving this method in order to characterize all tumour cells circulating in blood and apply it into the clinic in a real-rime liquid biopsy system.

#### 2.1.2. CTCs in Bladder Cancer

CTC detection in BC was first reported in 2000 by Lu et al., when they published a method for CTC detection in peripheral blood of patients with urothelial carcinoma using nested reverse transcription-PCR assay for *UPK2* (Uroplakin II) [75]. Their results were modest, being able to detect 3 out of 29 patients (10.3%) with superficial cancers (pTa-1N0M0), 4 out of 14 patients (28.6%) with MIBC (pT2-4N0M0), 2 out of 5 loco-regional node-positive patients (40.0%) (pN1-2M0) and 6 out of 8 patients (75.0%) with distant metastases [75].

More recently, several studies have evaluated CTCs in BC, mainly using CellSearch Technology, showing an average close to 50% of positive detection for metastatic BC and a low (around 15%) detection level for clinically localized BC [76]. Besides, even though CTC quantification has also been employed for prognosis and patient stratification, the detection of recurrent tumours is approximately 20–44% in patients showing progression upon recurrence. Additionally, Busetto and colleagues found a strong correlation between CTC presence and the time to first recurrence (75%) and they suggested that the time of progression is strongly correlated with CTCs [77].

In 2017, Zhang et al. published a meta-analysis of the impact of CTCs in BC. This study showed that the number of CTCs in peripheral blood is correlated with tumour stage, histological grade, metastasis and regional lymph node metastasis [45]. They also reported that the overall sensitivity and specificity of CTC detection assays are, respectively, 35% (95% CI: 28–43) and 97% (95% CI: 92–99), concluding that the presence of CTCs in peripheral blood is an independent predictive indicator of poor outcomes for urothelial cancer patients [44].

Even though CTC quantitation has to be studied more profoundly, this procedure could be incorporated into risk stratification algorithms and, therefore, aid patient management. In addition, CTC detection may not be accurate to be used as initial screening test but as a method for confirming BC diagnosis, due to the limited diagnostic sensitivity and high overall specificity. With improvements in clinical and laboratory techniques, the detection of CTCs at different time points in the future may allow real-time surveillance of dynamic changes of disease and crucially enhance our understanding of the metastatic cascade, thus facilitating novel targeted therapy approaches.

Regarding BC, the employability of CTCs in diagnosis and prognosis will be determined by the optimal combination of sensitivity, specificity, simplicity and cost of its implementation in the hospital routine. CTC enrichment techniques accompanied by a good cytological characterization may improve the fundamental weakness of cytology in the diagnosis/prognosis of low-grade disease. However, more well-designed, high-quality and large-scale prospective studies, especially including the CTCs and survival, are required to further strengthen current observations and shed more light on the potential of CTCs as a promising biomarker.

### 2.2. Circulating Cell-Free Tumour DNA (ctDNA)

#### 2.2.1. Detection and Genomic Analysis of ctDNA: First Clinical Approaches

As previously mentioned, the presence of cell-free DNA fragments in human blood was first discovered in 1948 [41]. In 1977, increased total cell-free DNA levels were observed in serum of cancer patients compared to healthy individuals, showing potential for therapeutic evaluation [78]. In blood, fragments of cell-free DNA have a typical size of 160–180 bp and are released from apoptotic as well as necrotic cells and possibly by active secretion, phagocytosis and exocytosis [40,79]. Methylation analysis has been used to trace the tissue of origin of cell-free DNA and showed that the biggest part in plasma is released by blood cells in healthy individuals [80]. At the end of the 1980s, Stroun et al. described that at least part of circulating free DNA in the plasma of cancer patients derived from cancer cells [81]. In 1991, DNA bearing *TP53* mutations were found in urinary sediments from MIBC patients, paving the way for the use of genomics in liquid biopsy [82]. Posteriorly, studies based on mutated *KRAS* sequences in plasma confirmed the tumour origin of mutant cell-free DNA [83]. Mutated genes in plasma were subsequently proposed to represent tumour markers and the term “circulating tumour DNA” was coined. On the other hand, ctDNA levels are very variable between individuals and the presence of metastasis as well as disease burden increase the heterogeneity of ctDNA levels [84]. In fact, the ctDNA fraction in plasma could represent up to 50% of all cell-free DNA in metastatic patients [85], whereas ctDNA may be undetectable in patients with MRD [86].

Despite these findings, poor technological advances have limited progress in this area for decades. For many years, multiple studies have been carried out to improve the detection systems that are used to observe tumour-associated genomic alterations in ctDNA, such as tumour-specific mutations, amplifications, deletions, gene rearrangements or methylation variations (Figure 2). These studies have tried to validate the potential of ctDNA as a diagnostic and prognostic marker in cancer as well as their value in MRD detection and therapeutic monitoring, mainly for patients with advanced malignancies [87,88,89,90,91]. However, the detection and quantification of ctDNA with a sensitivity required for significant clinical practice has not been easy, due to the small number of ctDNA fragments compared to the number of normal circulating DNA fragments.

Initially, allele-specific primers in conventional PCR and Pyrosequencing were used to detect and quantify the percentage of specific mutations in cell-free DNA present in liquid biopsy samples but the restriction to specific mutations as well as a low sensitivity (requiring, at least, a 10% of mutant DNA) has limited the success of these techniques [90,92]. This limitation in detection was improved by using quantitative PCR and different deep sequencing technologies such as NGS, being able to identify a 1–2% of mutations in different types of tumours [92,93,94,95,96,97]. Nowadays, digital droplet polymerase chain reaction (ddPCR) has improved accuracy and quantification of mutations, enabling more effective extraction and analysis of ctDNA, even in highly diluted cell-free DNA samples [98]. In 2005, Diehl and colleagues described for the first time the quantification of the mutant allele fraction of the *APC* gene in plasma of colorectal cancer patients by means of BEAMing technology, which is an approach based on digital PCR, binding to streptavidin beads, attachment of base pair-specific fluorescent probes and flow cytometry [99,100]. Both ddPCR and BEAMing have allowed the reduction of the detection limit of ctDNA mutations to 0.01–0.02% (Figure 2).

Despite the previously described technological advances, the abovementioned detection systems have some restrictions. Using PCR-based methods, the number of ctDNA alterations detected per assay is limited, only evaluating known and specific mutations. Besides, some techniques (like BEAMing) are laborious processes, keeping off a high productivity. Since the percentage of patients bearing known driver mutations is low, assays based on genome-wide analysis, which detection capacity has increased over the last years, have currently gained much importance. Newman et al. have developed a new system, called “cancer personalized profiling by deep sequencing (CAPP-Seq)” [101]. Here, they designed a multiple panel including somatic alterations from Catalogue Of Somatic Mutations In Cancer (COSMIC) and The Cancer Genome Atlas (TCGA) databases for non-small cell lung cancer, thereby detecting some of these alterations in 100% of high stage patients and in 50% of low stage patients, with a detection limit of approximately 0.02% [101] (Figure 2). Accordingly, these advanced techniques open a wide spectrum of possibilities to increase accuracy of diagnostic and predictive systems in a non-invasive form in cancer patients.

Worthy of note, the exact origin of ctDNA is not completely clear yet. Since ctDNA can be released from apoptotic or necrotic tumour cells which have died, genomic features derived from these cells may not entirely reflect the biology of primary tumours or metastasis at diagnosis, and, consequently, these alterations might not contribute to subsequent tumour progression and/or metastasis. This should also be taken into consideration during the clinical decision-making process.

#### 2.2.2. ctDNA in Bladder Cancer

Regarding ctDNA detection in BC patients, several studies have focused on the detection of different DNA alterations in liquid biopsy samples in order to find predictive biomarkers. In particular, urine has been proposed to be a bona fide liquid biopsy for diagnosis and prognosis of BC, given the proximity of tumours. The presence of ctDNA has been found in urine and plasma of BC patients and multiple studies have shown that high levels of ctDNA could be observed in urine of patients with progressive disease, even if ctDNA was not detected in plasma. These results support the usage of both plasma and urine liquid biopsy to detect BC, as well as to monitor recurrence and progression of the disease (reviewed in [86]).

As previously mentioned, *TP53* mutations in urinary sediments from invasive BC patients were described three decades ago [82]. Ever since, specific mutation hotspots in some genes, such as *PIK3CA*, *TERT*, *FGFR3*, *RAS* and *TP53*, have been targeted to detect mutations in ctDNA from BC patients, which has led to the discovery of associations between the presence of ctDNA mutations in these genes in urine as well as plasma samples and disease recurrence and progression [102,103,104]. Furthermore, using multiplex ligation-dependent probe amplification and NGS, copy number variations (CNVs) and mutations in tumour-related genes in plasma and urine of non-metastatic BC patients were identified, respectively. In this study, Patel et al. reported that the most common mutated genes were *TP53*, *KRAS*, *PIK3CA*, *BRAF*, *CTNNB1* and *FGFR3* and they found a loss of *CDKN2A* and *CREBBP* and gain of *E2F3*, *SOX4*, *PPARG*, *YWHAZ* and *MYCL1* [102]. The presence of some of these ctDNA mutations in plasma or urine (with a technical threshold of 0.5%) has been associated with early disease recurrence, achieving a sensitivity of 83% and specificity of 100% [102]. In plasma from MIBC patients, who show a high mutation rate, at least one mutation in the *PIK3CA*, *TP53* or *ARDIA1* hotspot regions or promoter region of *TERT* gene has been detected in 90% of the cases, as well as CNVs, observing *TP53* and *RB1* inactivating changes, *MDM2* gain or *CDKN2A* loss [85].

Moreover, loss of heterozygosity (LOH) has been shown by microsatellite-based PCR analysis in serum, plasma and urine of BC patients [69,105,106]. Microsatellite instability and LOH in liquid biopsy samples of BC patients are found relatively frequently using markers to detect alterations on chromosomes 4, 8, 9, 14 and 17 [105,106]. Chromosomal regions 17p and 9p are often affected in BC, disrupting the activity of tumour suppressor genes *TP53* and *CDKN2A*. This LOH seems to be associated with reduced disease-free survival and high risk of disease progression [107,108]. Since mutations and CNVs in ctDNA from plasma and urinary biopsies are detectable in high levels before progression, even in NMIBC patient and especially in urine samples, these biomarkers may be useful for disease monitoring [103]. Besides, some studies have revealed unknown alterations with differential sensitivity to therapeutic agents in metastatic patients, emphasizing the importance of ctDNA analysis as a useful tool for the detection of markers of therapy response and guidance of individualized therapies [86].

On the other hand, epigenetic alterations can be detected in BC patients using methylation -specific PCR (MSP) on ctDNA [69]. The combination of methylation levels of the *POU4F2* and *PCDH17* or *TWIST1* and *NID2* genes in urine samples showed a high capacity to differentiate BC patients from healthy volunteers, with 90% sensitivity and 93–94% specificity in both cases [109,110]. Dulaimi et al. reported the hypermethylation of *APC*, *RASSF1A* or *CDKN2A* (p14ARF) in urine ctDNA from 39 out of 45 BC patients (87% sensitivity and 100% specificity), even detecting 16 cases that showed a negative result in cytology assays [111]. Accordingly, hypermethylated DNA in urine of BC patients seems to be more common than positive cytology [111]. Besides, Hoque and colleagues described the combined methylation analysis of *CDKN2A*, *MGMT* and *GSTP1* using urine, enabling the differentiation between BC patients and control subjects, achieving 69% sensitivity and 100% specificity [112]. Furthermore, promoter methylation of both *CDKN2A* (p14ARF) and *MGMT* has been associated with tumour stage and the addition of *GSTP1* and *TIMP3* promoter methylation allowed to discriminate invasive tumours [112]. In cell-free serum DNA, hypermethylation of *APC*, *GSTP1* or *TIG1* has been shown to allow distinction between BC patients and control subjects with 80% sensitivity and 93% specificity [113]. Thus, the potential importance of methylation markers has been proposed for BC prevention and guidance of individual patient management in unpredictable BCs [114,115,116].

In addition to the alterations found in ctDNA, some commercial kits are based on DNA modifications present in exfoliated cells of the urine sediment. The UroVysion BC Kit is a multi-target FISH assay using exfoliated cells in urine that identifies aneuploidy of chromosomes 3, 7 and 17, as well as the loss of the 9p21 locus (which harbours tumour suppressor gene *CDKN2A*) [117]. A meta-analysis from 14 studies showed that the UroVysion kit has a diagnostic accuracy of 72% sensitivity and 83% specificity (AUC = 0.87) [42] (Table 1). Furthermore, based on methylation patterns of urine exfoliated cells, the 150 loci UroMark assay allows the detection of primary BC when compared to non-BC urine with a sensitivity of 98% and specificity of 97% (AUC = 0.97) [43] (Table 1).

### 2.3. Circulating Cell-Free RNAs

The presence of circulating cell-free RNA in liquid biopsy samples of cancer patients was described three decades ago, when alterations in the expression levels of some of them were observed in different types of cancer patients [118,119,120] and even associations with clinical outcome and disease prognosis were found [121,122,123,124]. Ever since, coding (mRNA) and non-coding (miRNA, lncRNA and piwi-interacting RNA) cell-free RNAs have gained much relevance as potential biomarkers in these sample types. Subsequently, principal studies related to each type of cell-free RNA in liquid biopsy in BC are described:

#### 2.3.1. Messenger RNAs

Circulating mRNAs were the first RNA molecules described in liquid biopsy in cancer patients [41]. Due to their intracellular role, cell-free mRNAs could be an important source of information about the status of activated or repressed signalling pathways into the tumour cells. Although a high percentage of these mRNAs are usually degraded by RNases, showing lack of stability and high variability between individuals [125,126,127], some mRNAs have demonstrated to have potential as biomarkers with diagnostic and predictive capacities.

With respect to total isoforms, the percentage of a full-length splicing variant of the *CA9* gene in urine sediments has shown to have diagnostic value to identify BC patients (AUC = 0.896) and this percentage was further increased in high grade and stage tumours [128]. Expression levels of *UBE2C* and *KRT20* mRNAs were significantly elevated in urine of urothelial cancer patients (sensitivity 82.5% and 85%; specificity 76.2% and 94.3%, respectively), increasing gradually with tumour grade and stage [129,130]. Bacchetti and collaborators observed significant differences in urine *PON2* expression when compared Ta and T1-3 tumours, showing higher expression in tumours confined to the basement membrane than in those invading other histological layers [131].

In order to improve the sensitivity and specificity of diagnostic and prognostic systems based on urine samples, several research groups have investigated different mRNA panels. Urquidi et al. carried out the combination of three different gene signatures [32,132,133] together with 6 other independent genes from different biomarker studies, after which they stablished a new diagnostic gene signature based on detected expression of 18 mRNAs (*ANXA10*, *BIRC5*, *CA9*, *CCL18*, *CDK1*, *CTSE*, *DSC2*, *IGF2*, *KFL9*, *KRT20*, *MDK*, *MMP1*, *MMP9*, *MMP10*, *MMP12*, *RAB1A*, *SEMA3D* and *SNAI2*) in urine samples from BC patients, achieving 85% sensitivity and 88% specificity (AUC = 0.935) [134]. Recently, the CxBladder Monitor and the Xpert Bladder Cancer Monitor (Table 1), two urine-based tests for BC surveillance which measure the expression levels of different sets of five mRNAs (*CDK1*, *CXCR2*, *HOXA13*, *IGFBP5* and *MDK*; and *ABL1*, *ANXA10*, *CRH*, *IGF2* and *UPK1B*, respectively), have been evaluated as follow-up methods for NMIBC patients after TURBT of primary or recurrent tumours. The CxBladder Monitor test was able to predict new recurrences after surgery with a sensitivity of 91% and a negative predictive value (NPV) of 96% (AUC = 0.73) [45,135], whereas the second test achieved a sensitivity of 84% and a specificity of 91% (AUC = 0.872) [47]. Biofina Diagnostics laboratory has developed a test based on ten differentially-expressed genes for the diagnosis and surveillance of BC from urine (UROBEST), achieving 80% sensitivity and 94% specificity (AUC = 0.91). Besides, the commercial laboratory Oncocyte has developed a panel of 43 gene expression biomarkers, PanC-Dx, to distinguish BC from non-cancerous conditions, showing good predictive values from urine samples (AUC = 0.91; sensitivity of 90% with a specificity of 82.5%) (Table 1) [48].

Besides instability and low abundance of circulating mRNAs, another problem associated to the use of mRNAs as biomarkers in liquid biopsy samples is the necessity of appropriate reference genes to compare the expression of target genes. Some studies have evaluated the expression of several potential housekeeping genes in urine samples, such as *PPIA*, *GAPDH*, *UBC*, *PGK1* and *ACTB* [132,136,137]. However, the potential of these and other genes as normalizers in biofluid samples should be studied more profoundly.

#### 2.3.2. microRNAs

Over the last decade, microRNAs have represented a type of biomolecules widely studied as biomarkers in different pathologies, including several types of cancers [138,139]. Moreover, miRNAs expression is very homogeneous among individuals, showing specific expression profiles in different types of tissue [140]. Additionally, miRNAs are protected by a protein complex and they are usually included in exosomes, thereby preserving their integrity and avoiding RNase-mediated degradation [141,142]. Due to these properties, miRNAs are very stable in liquid biopsy samples, such as serum, plasma and urine [137,143], which makes them potential candidates as biomarkers in non-invasive diagnostic and prognostic methods. On the other hand, the new systems designed to perform RT-qPCR from miRNAs allow the study of a wide number of miRNAs from very small amounts of total RNA.

In BC, multiple studies have identified individual miRNAs or panels with predictive features. Some of the most relevant studies of miRNAs in urine samples are discussed next. Downregulation of miR-145 allows to distinguish BC patients from healthy controls (77.8% sensitivity and 61.1% specificity for NMIBC, AUC = 0.729; 84.1% and 61.1% for MIBC, respectively, AUC = 0.790) and shows correlation with tumour grade [144]. Furthermore, miR-106b and miR-146a-5p have shown to be upregulated in BC patients, correlating with tumour stage and with grade and invasion, respectively [145,146]. In addition, high expression of miR-452 and miR-222 (with respect to the miR-16 expression level as normalizer gene) has shown to have diagnostic value (AUC = 0.848 and AUC = 0.718, respectively) [147] and the miR-126: miR-152 ratio has also enabled the detection of BC (AUC = 0.768) [148]. Upregulation of miR-214 has been associated with NMIBC patients but not with tumour grade or stage. Curiously, BC patients with lower levels of miR-214 presented a higher risk of recurrence [149]. Zhang et al. described that increased expression of miR-155 in urine is associated with tumour grade, stage, recurrence and invasion, allowing the discrimination of NMIBC patients, cystitis patients and healthy controls (80.2% sensitivity and 84.6% specificity) [150]. Besides, urine miR-200a has shown to have predictive properties, observing an association between low expression levels of this miRNA and high risk of recurrence in NMIBC patients [144]. Moreover, upregulation of miR-92a-3p and downregulation of miR-140-5p have been related to progression after recurrence [151].

On the other hand, there are some studies about miRNA expression in serum or plasma samples from BC patients, even though they are less frequent. High expression of miR-210 has been observed in serum of BC patients, correlating with tumour grade and stage and predicting progression (AUC = 0.898) [152]. In the case of plasma, expression of miR-19a is increased in tumour patients and associated with tumour grade [153], miR-200b is upregulated in MIBC, whilst miR-92 and miR-33 present inverse correlation with tumour stage [154].

Moreover, in the last years, several groups have developed multiple panels of miRNA expression, both in urine and in serum samples, to detect and monitor BC. Some of the main miRNA profiles are described in Table 2.

However, appropriate genes for normalization of miRNA expression in biofluids are unclear so far. In tissue samples, miRNA expression is usually normalized using the expression of small nuclear RNAs (snRNAs). Nevertheless, expression and stability of snRNAs is minimized in this type of sample [137], being inadequate as housekeeping genes. Although some authors have suggested some miRNAs, such as miR-16, miR-28-3p and miR-361-3p, as reference genes in urine samples [147,155], additional extensive studies are needed to determine specific housekeeping genes in the different types of liquid biopsies in this pathology. As observed for other tissues and disease conditions, specifically designed studies are required in order to find appropriate miRNAs, which do not show variation among the population to be separated (e.g., patients vs healthy controls, different disease state, metastatic vs non-metastatic disease), in serum and urine. In 2016, Martinez-Fernandez et al. described the use of two miRNAs, miR-193a and miR-448, as normalizers for urine studies [137]. However, these results still have to be validated in a well-designed clinical trial.

#### 2.3.3. Long Non-Coding RNAs

Long non-coding RNAs (lncRNAs) are transcripts longer than 200 nucleotides that are not translated into protein and can modify gene expression at transcriptional, post-transcriptional and epigenetic levels [156]. Although lncRNAs have not been as widely studied as mRNAs or miRNAs, multiple studies have shown that expression of these molecules can be altered in cancer, promoting tumour development, progression and metastasis [157] and, therefore, their use as biomarkers in biofluids is of growing interest.

*UCA1* (Urothelial cancer associated 1) is the most studied lncRNA in BC so far. Wang and collaborators determined that high expression of this lncRNA in urine sediments allows detection of high-grade superficial bladder tumours [158]. More recently, a meta-analysis of six studies, including 578 BC patients and 562 healthy controls, confirmed that upregulation of *UCA1* is able to predict BC (sensitivity of 81% and specificity of 86%, AUC = 0.88) [159]. Moreover, blood *UCA1* levels are upregulated in patients with metastatic BC after cisplatin treatment, increasing WNT6 protein expression and activating Wnt signalling, which results in cisplatin resistance [160]. Besides, overexpression of other lncRNAs, such as *HOTAIR*, *HOX-AS-2*, *MALAT1*, *HYMAI*, *LINC00477*, *LOC100506688* and *OTX2-AS1*, has been found in urine exosomes of high-grade MIBC patients [161]. On the other hand, other lncRNAs with biomarker potential are *ABHD11-AS1* and *H19* genes, whose increased expression has been associated with primary BC and early relapse, respectively, in tissue samples [162,163]. Future studies of these molecules in liquid biopsy samples of BC patients could be of great interest. 

In addition, expression of different types of cell-free RNAs can be combined to improve the accuracy of individual tests. Accordingly, Eissa and colleagues developed a panel from urine samples which combines the expression of one mRNA (*HYAL1*; Hyaluronoglucosaminidase 1), two miRNAs (miR-210 and miR-96) and one lncRNA (*UCA1*), thereby achieving a sensitivity of 100% and a specificity of 89.5% [169].

#### 2.3.4. Other Non-Coding RNAs and Its Future Potential as Biomarkers

Additionally, other non-coding RNAs, such as piwi-interacting RNAs (piRNAs) and circular RNAs (circRNAs) have been linked to BC. Although these molecules and their roles in cancer have been only recently studied, they could be good candidates as new biomarkers.

piRNAs are short single strands (26–31 nucleotides) of non-coding RNAs which can repress the expression of target genes, mediated by their binding to PIWI proteins (members of Argonaute proteins subfamily) [170]. Recently, several studies have reported that piRNAs can be widely detected in human plasma. Besides, the expression of some piRNAs has been found to be deregulated in patients with colorectal, prostate and pancreatic cancer [171,172]. Downregulation of piRNA DQ594040 has been associated with BC, whereas its overexpression can inhibit cell proliferation and promote cell apoptosis by upregulation of the TNFSF4 protein [173]. However, specific piRNAs have not yet been found in liquid biopsies from patients with BC.

circRNAs are a type of RNA which are covalently closed in a loop at the 3′ and 5′ ends. For this reason, these RNAs are more resistant than linear RNAs to degradation mediated by exonucleases and, therefore, show a prolonged half-life [174]. Although intra- and extra-cellular roles of these molecules are still largely unknown, some of them have shown relevance in several cancer types [175,176]. Overexpression of some circRNAs, such as circTCF25, circRNA-MYLK, circRNA-CTDP1 and circRNA-PC, has been observed in BC tissue samples. These circRNAs competitively bind to tumour suppressor miRNAs, acting as RNAs sponge and inhibiting their function [177,178,179]. Stability and functional properties of circRNAs make them interesting molecules to use as biomarkers in liquid biopsy samples.

### 2.4. Proteins and Peptides

The presence of proteins in liquid biopsy in cancer patients was first published in 1847 by Dr. Henry Bence Jones (reviewed in Reference [180]). Proteins and peptides (protein mass < 15 kDa) might be great candidates as biomarkers, since they are directly related to the “real-time” dynamic molecular cell phenotype. Nevertheless, the relevance of proteins and peptides as potential biomarkers in liquid biopsies has only been extensively studied over the last decade, due to limited technological advances. In BC, proteomic blood analyses [181,182] are scarce compared to the multiple studies performed with urine [53,132,169,183,184,185,186,187,188,189,190,191,192,193,194,195,196,197,198,199,200,201]. Plasma comprises the highly complex human-derived proteome, including the presence of a wide variety of proteins, which results in challenges with regard to detection and analysis systems. On the other hand, the urine proteome has been broadly studied and well-characterized, providing reference standards for data comparison and validation in the discovery of BC diagnostic markers [202].

#### 2.4.1. Peptide Biomarkers

In 2006, Theodorescu and colleagues have reported a diagnostic 22-peptide biomarker panel, using capillary electrophoresis coupled to mass spectrometry, which enables the differentiation between urinary BC patient samples and control samples (from prostate cancer, prostate hyperplasia, renal diseases and urinary tract infection), achieving 100% sensitivity and 73% specificity [195]. However, out of the 22 peptides, only fibrinopeptide A has been identified. Even though this peptide biomarker panel allows a good discrimination between advanced cancer and controls, less advanced tumours could not be correctly classified by this panel. Accordingly, the use of a predictive four polypeptide panel (fragments of membrane-associated progesterone receptor component 1, Collagen α-1 (I), Collagen α-1 (III) and Uromodulin) has been proposed as a relevant approach to distinguish between NMIBC and MIBC, reaching 92% sensitivity and 68% specificity [196]. Recently, the previously mentioned studies have been refined by Frantzi and collaborators, who discriminated two different panels using urine by performing a multi-centre study including 1357 patients. A 116-peptide biomarker panel (including identified Apolipoprotein A (APOA), β2-microglobulin, collagen fragments, fibrinogen A, Haemoglobin A, histidine-rich glycoprotein, insulin and small proline-rich protein 3) has been indicated for BC diagnosis, achieving 91% sensitivity and 68% specificity. The second panel has been proposed to encompass 106 peptide biomarkers (including identified ADAM22, ADAMTS1, Apolipoprotein A-1 (APOA-1), collagen fragments and HSPG2) allowing the detection of BC recurrences with 87% sensitivity and 51% specificity [197].

#### 2.4.2. Protein Biomarkers

Multiple proteomic studies have identified proteins (mass >15 kDa) and modifications with diagnostic and prognostic value in BC. However, a large variability has been observed between individual biomarker studies, reflecting proteomic complexity and the excess of applied proteomic approaches. Additionally, suboptimal experimental design of the individual studies contributes to inter-study inconsistency. Nevertheless, reproducible findings have also been reported in several independent studies. Some of the most relevant analyses are discussed next.

Differential expression of urinary α-1-antitrypsin (A1AT) has been indicated between BC patients and hernia patients (AUC = 0.729) as well as between BC patients and healthy controls (74% sensitivity and 80% specificity, AUC = 0.820) [198,199]. The upregulation of A1AT in patients with BC has subsequently been emphasized in an analysis by Linden and colleagues (66% sensitivity and 85% specificity) [200]. Besides, several studies have shown an increased abundance of apolipoprotein E (APOE) (89% sensitivity and 31% specificity, AUC = 0.745–0.756) and fibrinogen β (AUC = 0.720–0.831) in BC urinary biopsies compared to control patients [200,201]. Additionally, multiple studies have validated the importance of different apoliprotein types, reporting an increased abundance of APOA-1 in urine of BC patients (89–94.6% sensitivity and 85–92% specificity) compared to control patients, as well as an upregulation of APOA-2 (AUC = 0.631–0.864) in BC patients compared to hernia patients [183,198,201]. Besides, the differential expression of urinary carbonic anhydrase I and S100A8 between BC patients and hernia patients (AUC = 0.837 and AUC = 0.836, respectively) has been reported [198]. Moreover, Ebbing and colleagues have performed a study, including 181 samples from BC, prostate and renal cancer patients as well as healthy controls, in order to study the heterodimer S100A8/S100A9, known as calprotectin [184]. They showed a significant increase of calprotectin in BC biopsies (AUC = 0.880, 81% sensitivity and 93% specificity) compared to samples of the above-mentioned other tumour types and healthy controls [184]. In addition, Miyake et al. reported an increased abundance of COL4A1, COL13A1 and the combination of both collagens (COL4A1 + COL13A1) in BC urinary biopsies compared to healthy controls (sensitivity 68.2%, 54.6% and 72.1%; specificity 68.9%, 77.1% and 65.6%, respectively) [203]. Besides, the diagnostic sensitivity of this protein combination has been found to improve with malignancy grade, observing a value of 57.4% for low-grade tumours versus 83.7% for high-grade tumours [203].

The identification of biomarkers related to BC aggressiveness has been described by a limited number of studies. Zoidakis et al. performed a study including 108 BC patient samples and 97 urinary biopsies from control patients with benign disease (for example urolithisasis, benign prostate hyperplasia, infection/inflammation or haematuria) and found a differential expression of myeloblastin, aminopeptidase N and profilin-1 [185]. In addition, Nuclear interacting factor 1/Zinc finger 335 (NIF-1) and histone H2B have been described to be differently abundant in urinary biopsies from MIBC patients, NMIBC patients and benign controls [186].

Additionally, multiple analyses have been performed using protein panels, which might enhance accuracy in BC detection. In 2012, Goodison and colleagues proposed the use of a diagnostic 8-protein biomarker panel (angiogenin (ANG), APOE, CA9, IL8, matrix metallopeptidase 9 (MMP9), MMP10, plasminogen activator inhibitor 1 (PAI-1) and vascular endothelial growth factor A (VEGFA)) to distinguish BC patients and healthy controls in a study encompassing 127 urine biopsies, achieving 92% sensitivity and 97% specificity (AUC = 0.980) [187]. Additionally, Rosser et al. showed that a similar protein biomarker panel, namely the previously described 8-protein biomarker panel without CA9, enables the differentiation between BC patients and patients with different urological disorders (74% sensitivity and 90% specificity) [188]. Besides, Urquidi and collaborators reported a 3-protein biomarker panel (PAI-1, CD44 antigen and C-C motif chemokine 18 (CCL18)) to discriminate BC patients from healthy controls [204]. In 2014, Rosser et al. described the combination of 10 proteins (ANG, APOE, CA9, IL8, MMP9, MMP10, SDC1, Serpin Family A Member 1 (SERPINA1), Serpin Family E Member 1 (SERPINE1) and VEGFA) as a potential biomarker panel to detect recurrent disease in urine (79% sensitivity and 88% specificity) [189]. Two years later, Shimizu and colleagues published a comparable study using a similar protein panel (including PAI-1 and A1AT instead of SERPINA1 and SERPINE1) which allowed the differentiation of BC patients from benign and healthy controls, achieving 85% sensitivity and 81% specificity [190]. Recently, Soukap and collaborators reported the value of a 2-protein biomarker panel (synuclein G and midkine) combined with cytology in BC detection (91.8% sensitivity and 97.5% specificity) and showed that the addition of CEACAM1 and ZAG2 proteins to this panel enables the prediction of BC recurrences, achieving 92.7% sensitivity and 90.2% specificity [191].

As previously mentioned, only a limited number of plasma proteomic studies have currently been reported. Bansal et al. have proposed two differently expressed proteins, S100A8 and S100A9, distinguishing BC patients from healthy controls (AUC = 0.850–0.856 and AUC = 0.902–0.957) [181,182]. Moreover, in pre-operative compared to post-operative BC sera samples, a significantly increased abundance of annexin V was observed as well as a reduction of CA1, S100A4, S100A8 and S100A9 [181]. An upregulation of CA1 has also been observed in BC patients compared to healthy controls (AUC = 0.891–0.908) [181,182].

Overall, the previously described studies emphasize the diagnostic value of protein biomarker panels and individual protein biomarkers. However, their clinical value is still compromised due to suboptimal experimental design including benign or healthy controls (instead of clinically relevant patients) in many of the reported analyses, resulting in over-representation of BC. Nevertheless, some FDA-approved and non-approved diagnostic protein biomarkers are currently commercially available for clinical practice in BC (Table 1) and will be discussed next.

One of the most extensively studied proteins in BC urinary biopsies is Nuclear Matrix Protein 22 (NMP22) and multiple studies have demonstrated the use of this protein as a diagnostic BC biomarker, achieving 75–100% sensitivity and 75.9–91.8% specificity [169,192,193,194]. Mowatt and colleagues reported a pooled data analysis, encompassing a total of 13885 patients from 41 studies, showing that the performance of biomarker NMP22 exceeds cytology in BC detection with regard to sensitivity of the approach (68% versus 44%), mainly due to an improved detection of low-grade tumours [50,205]. Two assays, NMP22 BC test kit and NMP22 BladderChek Test, are currently in clinical use to detect NMP22 in urine. The NMP22 BC test kit represents the original approach based on a quantitative sandwich enzyme-linked immunosorbent assay (ELISA) test using two antibodies and has been FDA-approved for BC surveillance achieving 40% sensitivity and 99% specificity [49] (Table 1). On the other hand, the NMP22 BladderChek Test relies on a qualitative approach designed as a point of care (POC) analysis. The NMP22 BladderChek Test has been approved by the FDA for both BC surveillance and BC diagnosis (68% sensitivity and 79% specificity) [50] (Table 1). Grossman and collaborators analysed the clinical accuracy of the NMP22 BladderChek Test in two multi-centre studies [206,207], showing an increased sensitivity compared to cytology (56% versus 16%, respectively) in patients with haematuria but it did not reach the level of specificity obtained by cytology (86% versus 99%, respectively) [207]. Additionally, a combination of NMP22 BladderChek Test and cystoscopy has been observed to significantly enhance the detection of BC recurrence (up to 99%) compared to cystoscopy alone (91%) [206].

Next to NMP22, the bladder tumour antigen (BTA) has been approved by the FDA as a diagnostic biomarker in BC [208,209]. A pooled data analysis of 23 studies encompassing a total of 2258 BC patients and 2994 non-cancer individuals has shown that BTA allows for the differentiation of BC patients, achieving a mean sensitivity of 64% and specificity of 76.6% [53]. Two assays, BTA stat and BTA TRAK, have been developed for the detection of BTA in urine. BTA TRAK is an ELISA based approach, which has been approved for BC diagnosis, achieving 66% sensitivity and 65% specificity [51,210] (Table 1). BTA stat represents a qualitative assay for POC analysis, accepted for BC diagnosis with 70% sensitivity and 75% specificity [51,210] (Table 1). Besides, it has to be taken into account that multiple studies excluded patients with benign genitourinary conditions and including these patients would drastically diminish the BTA test specificity [211]. Therefore, this biomarker has currently limited clinical value.

On the other hand, cytokeratin fragment 21.1 (CYFRA 21.1) represents an ELISA test detecting soluble cytokeratin 19 fragments [52]. Multiple studies have reported that CYFRA 21.1 allows differentiation between liquid biopsies of BC patients and patients with non-cancer conditions, achieving 70–90% sensitivity and 73–86% specificity (AUC = 0.87–0.90) [52,53,212]. The specificity of this test dramatically decreases with the inclusion of patients with history of BCG and radiotherapy, excluding current use of the CYFRA 21.1 assay as a BC surveillance test [213,214].

Additionally, bladder cancer rapid test represents an urinary BC (UBC) test based on the detection of soluble fragments of cytokeratin 8 and 18, either using a quantitative ELISA or qualitative POC assay [215]. Multiple reports have shown that the UBC test enables the discrimination of BC patients compared to non-cancer individuals with a mean sensitivity of 64.4% and specificity of 80.3% [53,215]. Subsequently, Babjuk et al. described an increase in sensitivity (79%) as well as a decrease in specificity (49%) for the UBC test, once patients with benign conditions or other urinary tract malignancies were included [216]. In these cases, the BTA tests exceed the UBC rapid test regarding their use in BC detection [216].

### 2.5. Metabolites

The application of metabolomics in cancer is increasing over the years as this approach has shown importance in the search for candidate biomarkers. Since tumour cells are known to have altered metabolic pathways, metabolites in body fluids could be promising for the assessment of pathology, progression and prognosis of cancer [217]. Moreover, metabolomics has recently proved to be useful in the area of biomarker discovery for cancers in which early diagnostic and prognostic is urgently needed, such as BC. Given that the bladder is in intimate contact with urine, this body fluid has been mined heavily for metabolite biomarkers [218].

The use of metabolomic analysis in BC has been primarily focused on the distinction between normal-appearing urothelium and BC. Zhou et al. found a urinary four-biomarker panel (5-hydroxyvaleric acid, cholesterol, 3-phosphoglyceric acid and glycolic acid) including important metabolic characteristics (e.g., organic acid metabolism, steroid hormone biosynthesis, glycolysis and glyoxylate metabolism) and defined this panel as a combinatorial biomarker for the differentiation between BC patients and healthy controls (AUC = 0.804 with 78.0% sensitivity and 70.3% specificity in the validation set) [219,220]. Besides, Huang and colleagues reported the elevation of component I and decrease of carnitine C9:1 in BC urine samples, compared to healthy controls, as a promising biomarker panel for the identification of BC patients (92.6% sensitivity and 96.9% specificity; AUC = 0.963) [221]. However, the structure and biological function of component I is still unclear and required to be studied as it has not been previously observed in nature. Nevertheless, carnitines are an example of disturbed fatty acid transportation, fatty acid-oxidation, or energy metabolism that is happening in tumour cells [221]. Supporting these findings, Ganti proposed that acylcarnitine appearance in BC patient urine samples varies widely in function of tumour grade, suggesting that consistently lower levels of acylcarnitines are present in the urinary biopsies of BC patients with low grade tumours as compared to both BC patients with high grade tumours as well as healthy controls [222]. These results have raised the possibility that fatty acid abnormalities might be involved in the pathogenesis of the tumour.

Moreover, Sahu and colleagues confirmed unique pathway alterations that differentiate MIBC and NMIBC [223]. MIBC appears to preferentially enhance cyclooxygenase (COX) and lipoxygenase (LOX) signalling (Eicosanoids, prostaglandins and tromboxanes (*p*-value < 0.004), increase heme catabolism (*p* = 0.0001) and alter nicotinamide adenine dinucleotide (NAD+) synthesis (kynurenine (*p* = 0.0212), anthranilate (*p* = 0.0111) and quinolate (*p* = 0.0015)) [223] with a possible influence in inflammatory cell regulation, cell proliferation and angiogenesis [224,225]. Supporting these results, Loras and colleagues were recently able to identify metabolites in urine enabling the discrimination of BC patients with a high sensitivity (87.9%) and specificity (100%) and a negative likelihood value of 0.1, as well high negative predictive values for low, low-intermediate and high-intermediate and high-risk patients [226]. Metabolomic analysis revealed altered phenylalanine, arginine, proline and tryptophan intermediate metabolism associated to NMIBC [226]. These studies suggest that different stages/grades of BC might generate distinct metabolic profiles, which might be due to the fact that cancer cells in advanced grades/stages require more energy for survival and continuous growing.

Next to the use of urinary analysis for the identification of metabolites as possible biomarkers, the evaluation of global serum profiles of BC, kidney cancer and non-cancer controls has revealed potential biomarkers for BC, including eicosatrienol (AUC = 0.98), azaprostanoic acid (AUC = 0.977), docosatrienol (AUC = 0.972), retinol (AUC = 0.801) and 14′-apo-beta-carotenal (AUC = 0.767) [227].

Overall, the BC metabolic signature is mainly characterized by alterations in metabolites related to energy metabolic pathways, amino acid and fatty acid metabolism, which are known to be crucial for cell proliferation as well as glutathione metabolism, a determinant in maintaining cellular redox balance [228]. However, the absence of a standard for sample acquisition, use of different platforms to profile metabolites, environmental stress and food intake strongly influence the composition of the metabolome and all these factors have led to a large diversity of metabolomic profiles obtained from different laboratories. These issues need to be considered, since they heavily affect the quality of the results by introducing bias and artefacts. Nevertheless, despite remaining challenges, metabolomics shows great clinical promise. The improved sensitivity, specificity of technics and the development of an in-depth reference metabolome may help to identify good metabolic biomarkers which can eventually be translated into the clinic.

### 2.6. Extracellular Vesicles

The concept of extracellular vesicles (EVs) has evolved from being considered garbage bags to the demonstration that extracellular vesicles could play very interesting roles and functions in cancer biology by promoting survival and growth of disseminated tumour cells; enhancing invasiveness; promoting angiogenesis, migration, tumour cell viability and inhibiting tumour cell apoptosis [229,230]. EVs include microvesicles, apoptotic bodies and exosomes, with the latter being mostly studied at present. Therefore, in this review, we will mainly focus on the potential of exosomes as cancer biomarkers in BC.

Exosomes are small (30–100 nm) membrane vesicles released into the extracellular environment due to fusion of multivesicular bodies with the plasma membrane. They were first described in 1983 in two different papers, published simultaneously [231,232] and currently tumour-released microvesicles, which are abundant in the body fluids of patients with cancer, are suggested to be involved in tumour progression [233]. Besides, it has been demonstrated that exosomes may help in immune response modulation, presentation of antigens to immune cells and intercellular communication through transfer of proteins, mRNAs and miRNAs, which could be a useful tool for diagnostic, predictive and prognostic purposes in different types of tumours. Regarding this, Valenti and colleagues showed that another kind of EVs, microvesicles, released by human melanoma and colorectal carcinoma cells, can promote the differentiation of monocytes to myeloid-derived suppressor cells, which support tumoral growth and immune escape [234].

Currently, there is an increasing interest in the application of exosomes as non-invasive cancer biomarkers and many studies have demonstrated that molecules, such as the lncRNAs *HOTAIR*, *HOX-AS-2*, among others and proteins, like EDIL3 and periostin, are significantly altered in patients with BC [161]. Therefore, EVs are proposed to be enriched in proteins that can be associated with signalling pathways related to tumorigenesis. In this way, Silvers reported that EVs collected from urine of six BC patients (pT1-pT3) showed, at least, a fifteen fold enrichment in the protein levels of β-Hexosaminidase (HEXB), S100A4 and Staphylococcal nuclease and tumour domain containing 1 (SND1) compared to the urinary protein levels of six healthy volunteers (*p* < 0.05) [235]. However, despite these promising preliminary results, the size of this study population is confined and additional extensive research is required for the validation of these data.

Furthermore, based on their stability in body fluids, especially exosomal miRNAs are discussed to be useful diagnostic and prognostic biomarkers in liquid biopsies. Baumgart and colleagues showed that exosomes from invasive BC cell lines, compared to non-invasive BC cell lines, are characterized by a specific miRNA signature which could play a role in the modification of the tumour microenvironment (*p* < 0.05; FC > 1.5) [236]. These results confirmed the hypothesis that the molecular content of exosomes is, at least in part, similar to that of host cells and reflects their cellular properties. However, Baumgart also analysed urinary exosomes from BC patients and they exhibited only in part the miRNA alterations detected in cell line exosomes [236]. Therefore, further analyses will have to clarify the functional relevance of exosomal miRNAs and their role as molecular markers in liquid biopsies.

Even though EVs are a promising source of cancer biomarkers, few studies have been done and no exosomal biomarkers have been implemented in BC clinical practice so far. In general, the interest in EVs is growing but the introduction as established predictive biomarkers has been hampered by challenges in exosome isolation and characterization, indicating the need for new sensitive platforms which allow more accurate isolation and detection methods. Furthermore, the use of an efficient, rapid and reproducible isolation method is fundamental for analytical reproducibility.

## 3. Summary and Discussion

Among body fluids, urine and saliva are the most attractive fluids for liquid biopsy due to their accessibility and low invasiveness of collection. As somatic alterations detected in ctDNA are reflective for those present in tumour tissue, the ctDNA profile could be a practical method for obtaining the tumour genome independently of direct tissue sequencing. Additionally, mutations in ctDNA of cancer patients could be detected over one year prior to clinical diagnosis, which emphasizes the great potential of liquid biopsy for the detection of cancer at early stages [98,237,238]. At present, there are several diagnostic kits based on the detection of mutations in liquid biopsy samples using ctDNA or CTCs from the bloodstream. Most of them have been designed for blood/plasma/serum samples using qPCR and NGS techniques. Only the diagnostic kit Trovera (initially designed for the identification of mutations in *BRAF*, *KRAS*, *EGFR* in plasma samples; Trovagene) is marketed for both plasma and urine samples. A diagnostic alternative is based on the detection of both circulating RNA and extracellular vesicles (such as exosomes), for which multiple diagnostic kits are brought on the market in order to detect and monitor prostate (like ExoDx Prostate; IntelliScore) or bladder (like CxBladder; Pacific Edge, among others) cancer in urine samples.

**Figure 3:**
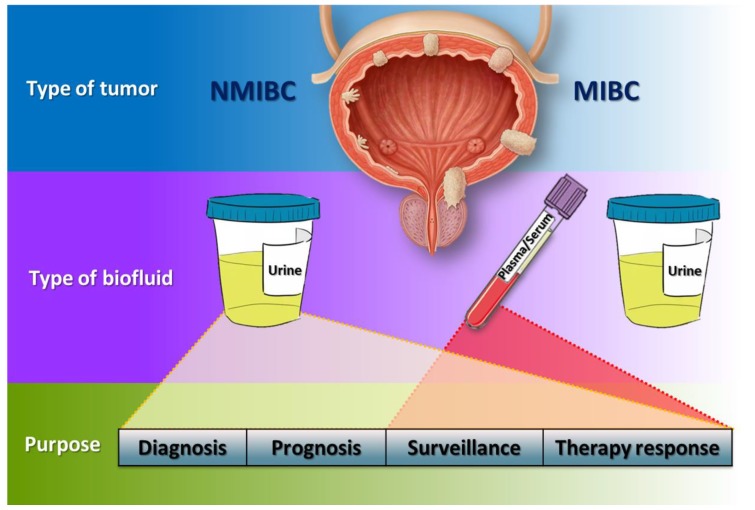
Hypothetical flowchart of liquid biopsies management in BC. In NMIBC patients, urine could be the best type of biofluid for diagnosis, prognosis, surveillance and therapy response due to its intimate contact with the tumour, whilst in MIBC patients, though urine could also be used, plasma and serum acquire more importance to monitor patients.

Although it is true that urine can reflect genetic alterations of a large number of solid tumours [239], it will probably be more relevant for the diagnosis and monitoring of tumours of the genitourinary tract. In these cases, the content of nucleic acids from the tumour cells is released directly into the urine, which minimalizes the DNA/RNA contamination background of blood cells as observed in plasma (Figure 3) [240].

As previously mentioned, the high recurrence rate and the need for expensive diagnostic and monitoring methods, such as cystoscopy, make BC the most expensive human cancer from diagnosis to death. For this reason, efforts to develop diagnostic, prognostic and follow-up systems for BC have been enormous in recent years, with various systems published for liquid biopsy samples. Accordingly, several diagnostic laboratories have launched different diagnostic and monitoring systems for BC patients, which are based on the determination of gene expression or protein biomarkers in urine samples (Table 1). Moreover, the identification of metabolites as potential biomarker in BC liquid biopsy has also been explored. Several authors have found specific metabolites that are able to identify patients with BC, even before appearance of the first clinical symptoms of this disease [241]. However, the main concern regarding metabolomics in urine as a diagnostic system is the variability of glomerular filtration, both with medication and dietary habits as the main confounding factors [242,243]. Therefore, large cohort studies and standardization of sample taking and processing procedures will be necessary to finally establish metabolomics as a diagnostic approach.

Regarding MIBC patient follow-up, it should be taken into account that, even though cystectomy is performed in most cases, progression of bladder tumours is produced by metastasis in other tissues and organs. In case of metastatic tumours, blood becomes perhaps the most appropriate fluid for follow-up and to explore possible therapies once progression of the disease is established (Figure 3). Therefore, the determination of mutations or alterations of gene expression patterns has been explored from both ctDNA in plasma/serum and from the isolation of CTCs in the bloodstream (reviewed in [244,245,246]). However, care must be taken with predictions regarding the future of these new technologies and the studies that support them. Some of the current FDA-approved systems for the diagnosis and monitoring of BC do not meet sensitivity and specificity requirements (e.g., the NMP22 determination), whereas other tests have such high costs that their use in daily health practice is limited (e.g., the UroVysion test). Consequently, there is an urgent need for suitable studies in order to validate biomarkers for early detection. Nevertheless, conventional case-control studies have proven not to be adequate, emphasizing the importance of prospective cohort studies, consisting of serial samples at different time points from a person at-risk, as well as large randomized trials, validating biomarker clinical benefit compared to actual gold standard methods. Additionally, a coherent and comprehensive set of guidelines must be delineated to ensure success once an approach is approved for clinical set-up. For example, Pepe et al. described a prospective randomized open blinded end-point (PROBE) study design which takes into account components related to the clinical context and outcomes, criteria for measuring biomarker performance, the biomarker test itself and the size of the study as a guidance for the design of a biomarker accuracy study [247]. Besides, sample repositories (crucial for the discovery and evaluation of biomarkers with potential use in clinical medicine) should follow this design strategy in order to maximize biomarker values.

## 4. Concluding Remarks

In general, physicians and researchers agree that liquid biopsy is the most promising strategy for diagnostics, selection of treatments and follow-up in various tumour types. However, it is important that the development of these new diagnostic and follow-up systems come together with the appropriate proposals for changes in the therapeutic procedure, either with a better characterization of the patients or with an adequate proposal of an effective treatment line. On the other hand, the lack of validation of these systems, which are capable of detecting a tumour burden much smaller than the imaging technologies, currently prevents them from clinical practice, since they can generate great anxiety among patients and possibly lead to overtreatment of the patient. Therefore, more studies with long follow-up periods and large cohorts are required to demonstrate that the positive result in a liquid biopsy test is valid as a starting point to initiate or change an oncologic treatment. However, despite the difficulties and current limitations in liquid biopsy technologies and the current lack of robust and confident methodologies that unequivocally allow diagnosis, prognosis or detection of therapy response, with the current accumulation of clinical evidence, we are convinced that it will only be a matter of time until liquid biopsy replaces tissue biopsy in all solid tumours.

## Figures and Tables

**Table 1:** Commercial kits to detect and follow-up bladder cancer (BC) using liquid biopsy biomarkers.

Commercial Kits	Biomarker	Assay Type	Sample Type	FDA Approved	Purpose	Predictive Capacity	Source	Refs.
Cytology	Sediment cells	Giemsa and HE staining	Urine	Yes	Diagnostic and surveillance (1)	Sensitivity = 38% Specificity = 98%	-	[26]
uCyt+	Sediment cells	Immunofluorescence	Urine	Yes	Surveillance in adjunct to cystoscopy	Sensitivity = 73% Specificity = 66%	DiagnoCure (2)	[29]
UroVysion	Sediment cells	Multi-target FISH	Urine	Yes	Diagnostic	Sensitivity = 72% Specificity = 83%	Abbott	[42]
UroMark(3)	Sediment cells	Bisulfite-based methylation assay	Urine	No	Diagnostic	Sensitivity = 98% Specificity = 97%	Kelly:Feber	[43]
CellSearch	CTCs	Immunomagnetic enrichment	Plasma/serum	Yes	Surveillance	Sensitivity = 48% Specificity = 98%	Menarini-Silicon Biosystems	[44]
CxBladder	mRNA	RT-qPCR	Urine	No	Diagnostic	Sensitivity = 82% Specificity = 85%	Pacific Edge	[32]
CxBladder Monitor	mRNA	RT-qPCR	Urine	No	Surveillance	Sensitivity = 91% NPV = 96%	Pacific Edge	[45]
Xpert BC Detection	mRNA	RT-qPCR	Urine	No	Diagnostic	Sensitivity = 76% Specificity = 85%	Cepheid	[46]
Xpert BC Monitor	mRNA	RT-qPCR	Urine	No	Surveillance	Sensitivity = 84% Specificity = 91%	Cepheid	[47]
PanC-Dx	mRNA	RT-qPCR	Urine	No	Diagnostic	Sensitivity = 90% Specificity = 83%	Oncocyte	[48]
UROBEST (4)	mRNA	RT-qPCR	Urine	No	Diagnostic and surveillance (5)	Sensitivity = 80% Specificity = 94%	Biofina Diagnostics	-
NMP22	Protein	Sandwich ELISA	Urine	Yes	Surveillance	Sensitivity = 40% Specificity = 99%	Abbott	[49]
NMP22 BladderChek	Protein	Dipstick immunoassay	Urine	Yes	Diagnostic and surveillance (1)	Sensitivity = 68% Specificity = 79%	Abbott	[50]
BTA TRAK	Protein	Sandwich ELISA	Urine	Yes	Diagnostic and surveillance (1)	Sensitivity = 66% Specificity = 65%	Polymedco	[51]
BTA stat	Protein	Dipstick immunoassay	Urine	Yes	Diagnostic and surveillance (1)	Sensitivity = 70% Specificity = 75%	Polymedco	[51]
CYFRA 21.1	Protein	Immunoradiometric assay or ELISA	Urine	No	Diagnostic	Sensitivity = 82% Specificity = 80%	CIS Bio International	[52]
UBC test	Protein	Sandwich ELISA or dipstick immunoassay	Urine	No	Diagnostic	Sensitivity = 64% Specificity = 80%	IDL Biotech	[53]

(1) Although these tests have been proposed for diagnosis and follow-up of BC, predictive values correspond to the detection of primary tumour. (2) DiagnoCure company was dissolved in 2016 and the uCyt+ test is not available at present. (3) The performance of the UroMark test is currently evaluated in Phase III studies. (4) UROBEST is not yet commercially available. (5) Biofina Diagnostics provides these predictive values for diagnostic and surveillance purposes together. NPV = Negative predictive value.

**Table 2:** Main miRNA panels for diagnosis, prognosis and recurrence surveillance of BC using liquid biopsy samples.

Studies [References]	Type of Sample	Clinical Application	miRNA Panels	Predictive Capacity
Sapre N. [164]	Urine	Recurrence surveillance	miR16, miR200c, miR205, miR21, miR221 and miR34a	Sensitivity = 88%
				Specificity = 48%
				AUC = **0.74**–0.85
Pardini B. [155]	Urine	Diagnostic and prognosis	NMIBC G1 + G2 *: miR-30a-5p, let-7c-5p, miR-486-5p, miR-205-5p and let-7i-5p	AUC = 0.73
			NMIBC G3 *: miR-30a-5p, let-7c-5p, miR-486-5p, miR-21-5p, miR-106b-3p, miR-151a-3p, miR-200c-3p, miR-183-5p, miR-185-5p, miR-224-5p, miR-30c-2-5p and miR-10b-5p	AUC = 0.95
			MIBC *: miR-30a-5p, let-7c-5p, miR-486-5p, miR-205-5p, miR-451a, miR-25-3p, miR-30a-5p and miR-7-1-5p	AUC = 0.99
Jiang X. [165]	Serum	Diagnostic	miR-152, miR-148b-3p, miR-3187-3p, miR-15b-5p, miR-27a-3p and miR-30a-5p	AUC = 0.899
Jiang X. [166]	Serum	Prognosis	MIBC: miR-422a-3p, miR-486-3p, miR-103a-3p and miR-27a-3p	AUC = **0.880**-0.894
Du L. [167]	Urine	Diagnostic	miR-7-5p, miR-22-3p, miR-29a-3p, miR-126-5p, miR-200a-3p, miR-375 and miR-423-5p	Sensitivity = 82–**85%**
				Specificity = **87**–96%
				AUC = **0.916**–0.923
Urquidi V. [168]	Urine	Diagnostic	miR-652, miR-199a-3p, miR-140-5p, miR-93, miR-142-5p, miR-1305, miR-30a, miR-224, miR-96, miR-766, miR-223, miR-99b, miR-140-3p, let-7b, miR-141, miR-191, miR-146b-5p, miR-491-5p, miR-339-3p, miR-200c, miR-106b *, miR-143, miR-429, miR-222 and miR-200a	
				Sensitivity = 87%
				Specificity = 100%
				AUC = 0.982


* Including traditional BC risk factors (age and smoking status). Bold numbers indicate values from validation set.
